# l-leucine partially rescues translational and developmental defects associated with zebrafish models of Cornelia de Lange syndrome

**DOI:** 10.1093/hmg/ddu565

**Published:** 2014-11-06

**Authors:** Baoshan Xu, Nenja Sowa, Maria E. Cardenas, Jennifer L. Gerton

**Affiliations:** 1 Stowers Institute for Medical Research, Kansas City, MO 64110, USA,; 2 Department of Biochemistry and Molecular Biology, University of Kansas School of Medicine, Kansas City, KS 66160, USA,; 3 Medical Faculty, University of Göttingen, Robert-Koch-Str. 40, 37075 Göttingen, Germany; 4 Department of Molecular Genetics and Microbiology, Duke University, Durham, NC 27708, USA

## Abstract

Cohesinopathies are human genetic disorders that include Cornelia de Lange syndrome (CdLS) and Roberts syndrome (RBS) and are characterized by defects in limb and craniofacial development as well as mental retardation. The developmental phenotypes of CdLS and other cohesinopathies suggest that mutations in the structure and regulation of the cohesin complex during embryogenesis interfere with gene regulation. In a previous project, we showed that RBS was associated with highly fragmented nucleoli and defects in both ribosome biogenesis and protein translation. l-leucine stimulation of the mTOR pathway partially rescued translation in human RBS cells and development in zebrafish models of RBS. In this study, we investigate protein translation in zebrafish models of CdLS. Our results show that phosphorylation of RPS6 as well as 4E-binding protein 1 (4EBP1) was reduced in *nipbla/b*, *rad21* and *smc3*-morphant embryos, a pattern indicating reduced translation. Moreover, protein biosynthesis and rRNA production were decreased in the cohesin morphant embryo cells. l-leucine partly rescued protein synthesis and rRNA production in the cohesin morphants and partially restored phosphorylation of RPS6 and 4EBP1. Concomitantly, l-leucine treatment partially improved cohesinopathy embryo development including the formation of craniofacial cartilage. Interestingly, we observed that alpha-ketoisocaproate (α-KIC), which is a keto derivative of leucine, also partially rescued the development of *rad21* and *nipbla/b* morphants by boosting mTOR-dependent translation. In summary, our results suggest that cohesinopathies are caused in part by defective protein synthesis, and stimulation of the mTOR pathway through l-leucine or its metabolite α-KIC can partially rescue development in zebrafish models for CdLS.

## Introduction

Cohesin is a protein ring structure that holds sister chromatids together from DNA replication until cell division. The cohesin complex is composed of four subunits: Scc1 (Rad21/Mcd1), Scc3, Smc1 and Smc3. Smc1 and Smc3 are family members of Structural Maintenance of Chromosomes proteins. Scc1 and Scc3 interact with the ATPase-containing head domains of Smc1 and Smc3 to stabilize the cohesin ring structure. Cohesin facilitates chromosome segregation, DNA damage repair and gene expression. Reduced cohesin function can cause the differential expression of many genes. The molecular mechanisms behind cohesin's regulation of gene expression are still elusive but are critical for animal development. Cohesin is physically associated with several transcriptionally active regions within the metazoan genome, such as the H19 imprinting control region (1) and the β-globin locus control region (2), suggesting cohesin plays a critical role in gene expression.

Mutations of cohesin subunits as well as its regulators lead to a spectrum of disorders known as cohesinopathies which include both Cornelia de Lange syndrome (CdLS) and Roberts syndrome (RBS). Characteristics of cohesinopathies can include poor growth, limb, gut and heart defects, craniofacial abnormalities and mental retardation ranging from mild to severe. Mutation of both copies of Establishment of Sister chromatid Cohesion acetyltransferase 2 (ESCO2) causes RBS (3). CdLS is a clinically distinct syndrome caused by mutation in one of the two copies of a number of different genes, suggesting the syndrome is caused by haploinsufficiency for cohesin function. Fly Nipped-B-like protein (NIPBL) is a homolog of fungal Sister Chromatid Cohesin protein (*SCC2*) that loads the cohesin complex onto the chromosome. The cohesin loading factor was first reported to be mutated in CdLS in 2004 (4,5). Around 60% of CdLS cases are caused by an NIPBL mutation. Smc1 and Smc3 were subsequently found to be mutated in a small number of CdLS patients with mild symptoms (6,7). Recently, HDAC8 mutations were documented in several CdLS patients (8). HDAC8 deacetylates the Smc3 subunit of cohesin (8). A related cohesinopathy, which has overlapping features with CdLS, is caused by mutations in Rad21 (9). Therefore, mutations in many different cohesin-related genes can cause developmental disorders with both overlapping and unique features. Cells derived from CdLS patients do not show high levels of aneuploidy (10), consistent with the hypothesis that changes in gene expression cause this syndrome.

Whole animal models have been developed to study pathogenesis in the cohesinopathies. A CdLS mouse model (Nipbl+/−) mimics many features of CdLS including small size, craniofacial anomalies, heart defects and perinatal mortality, along with dysregulated expression of many genes across various tissues (11). Multiple studies performed in zebrafish found that *rad21*-morphant and *rad21* transgenic mutant embryos displayed central nervous system necrosis, decreased head and eye size and decreased trunk thickness (9,12,13). There are two copies of *nipbl* in the zebrafish genome; the *nipbla/b*-knockdown embryos exhibited disrupted blood circulation, defective cardiac precursor migration and decreased gut thickness (14). The *smc3*-morphant embryos have brain and eye necrosis (15). The *esco2* and *rad21* morphant embryos demonstrated mitotic delay and elevated apoptosis (12,16), both of which may contribute to the developmental abnormalities.

Two major regulatory proteins are affected in zebrafish cohesin morphant embryos—p53 and c-Myc. p53, a tumor suppressor protein and cell cycle regulator, is upregulated in *rad21*, *smc3* and *esco2* morphant zebrafish embryos (14–17). There are many different triggers for p53, including defects in ribosome biogenesis. It is unclear which triggers cause the upregulation observed in cohesin morphant zebrafish. The cohesin complex physically binds to the promoter of c-Myc, which plays an important role in cell growth and apoptosis and facilitates transcription of many genes involved in translation. In contrast to p53, c-Myc is downregulated in *rad21, nipbla/b and smc3*-morphant zebrafish embryos as well as in *D. melanogaster* mutants (13), but upregulated in *esco2* morphants. As c-Myc is a positive regulator of ribosome biogenesis and protein synthesis (18) and p53 serves as a cellular sensor for ribosome impairment, then the upregulation of p53 in combination with the downregulation of c-Myc suggests that ribosome function and protein translation could be compromised by reduction of *rad21* and *smc3* activity.

We wondered whether CdLS would be associated with defects in translation, similar to RBS. Cohesin is enriched at the ribosomal DNA (rDNA) repeat regions in both prokaryotes and eukaryotes. These repeats are located in the nucleolus and highly transcribed by RNA polymerase I to make ribosomal RNAs that are structural components of the ribosome. Cohesin likely influences not only RNA polymerase II transcription but also rRNA production and ribosome function, which are vital for development and cell growth. Cells derived from individuals with RBS displayed highly fragmented nucleoli, and defects in ribosome biogenesis and protein translation, similar to yeast with a mutation in the cohesin acetyltransferase (17,19). Changes in gene expression associated with mutations in cohesin may be derived from direct effects at individual genes, but also indirect effects caused by the misregulation of key pathways and processes, such as mTOR (mammalian target of rapamycin), nucleolar function and protein synthesis. l-leucine stimulation of the mTOR pathway partially rescued translation in human RBS cells and development in zebrafish models for RBS. A few reports have shown that the l-leucine metabolite alpha-ketoisocaproate (α-KIC), which is a keto derivative of leucine made by branched chain aminotransferases BAT1 and BAT2 enzymes (20), is also able to activate the mTOR pathway, similar to l-leucine (21,22). If reduced cohesin function is associated with defective translation, we further hypothesized that α-KIC or l-leucine might rescue development in zebrafish models for CdLS.

In this study, we investigated protein translation in zebrafish models for CdLS. We measured two indicators of translation downstream of the mTOR kinase, the phosphorylation of RPS6 and 4E-binding protein 1 (4EBP1). The phosphorylation of RPS6, which is a component of the 40S ribosomal subunit, promotes translation. The phosphorylated form of eukaryotic translation initiation factor 4EBP1 disassociates from eIF4E to promote translation initiation. We found that CdLS zebrafish morphants showed low levels of phosphorylation of both RPS6 and 4EBP1, consistent with translation inhibition. Furthermore, metabolic labeling experiments indicated that rRNA production and protein synthesis were low compared with controls. Given the hypothesis that CdLS may be due in part to poor translation function, we tested the effect of l-leucine and α-KIC on CdLS zebrafish embryos. l-leucine and α-KIC boosted RPS6 and 4EBP1 phosphorylation, improved survival, protein synthesis and rRNA production and partially rescued several aspects of development in zebrafish models of CdLS. Taken together, our results strongly suggest that translational defects contribute to the developmental phenotypes associated with reduced cohesin function in zebrafish and targeting translation could be a useful therapeutic strategy for CdLS.

## Results

### Zebrafish cohesinopathy models display translational inhibition

To examine the molecular signatures and developmental defects in animal cohesinopathy models, we utilized cohesin morphant zebrafish embryos. Morphants (MO) were created by microinjecting zebrafish embryos (1–2 cells) with a morpholino to knockdown *rad21*, *smc3* and *nipbla/b* as previously described (13–15). The morpholinos were injected into the center of the yolk to avoid the chance of secondary effects owing to mechanical damage (23). Control morpholinos included a morpholino with 5-base mismatches (*nipbla/b-*5mis) and a random control oligo 25-N (*ctrl*-MO) from Gene Tools, LLC. The dose of morpholino for each gene was initially titrated to reduce lethality at early stages of development (not shown). For the dose chosen for experiments, we tested the degree of knockdown by immunoblotting analysis of Rad21, Smc3 and Nipbla/b protein (Supplementary Material, Fig. S1). For Smc3, the morpholino reduced the protein level by ∼50% whereas the Rad21 and Nipbla/b morpholino reduced the protein level by ∼70% at 1 day post-fertilization (dpf). After this time, embryos do not lyse well, precluding western blot analysis. The *ctrl*-MO-injected embryos did not show any phenotypic changes compared with uninjected wild-type (WT) embryos.

**Figure 1.**
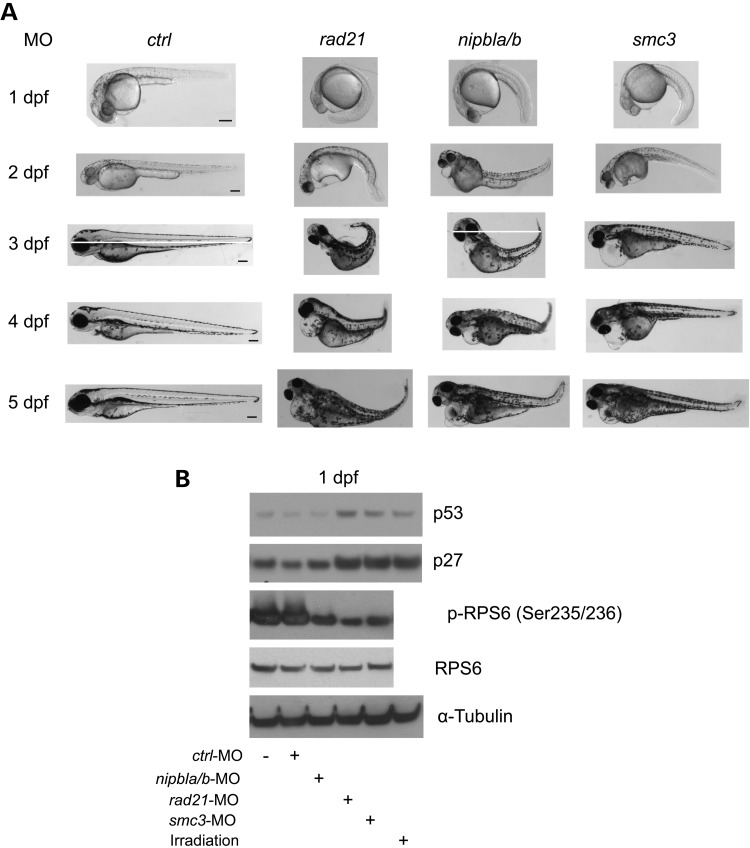
Cohesin morphant embryos display a spectrum of developmental defects, and a molecular signature of impaired translation. (**A**) Embryos were microinjected with Random control morpholino (*ctrl*-MO), *nipbla/b* morpholino (*nipbla/b*-MO), *rad21* morpholino (*rad21*-MO) or *smc3* morpholino (*smc3*-MO). Embryos were imaged each day up to 5 days. Developmental defects of cohesin morphant embryos were apparent from 1 to 5 days. Scale bar = 200 µm. (**B**) Embryos were microinjected as in (A). In addition, uninjected embryos and embryos subjected to ionizing radiation (10 Gy irradiation, 12-min exposure) were collected as a negative and positive control for p53 levels, respectively. After 1 dpf, we performed western blot analysis of p53, p27, and translation component RPS6. The blots were quantified with ImageQuant TL software (Supplementary Material, Fig. S2A).

**Figure 2.**
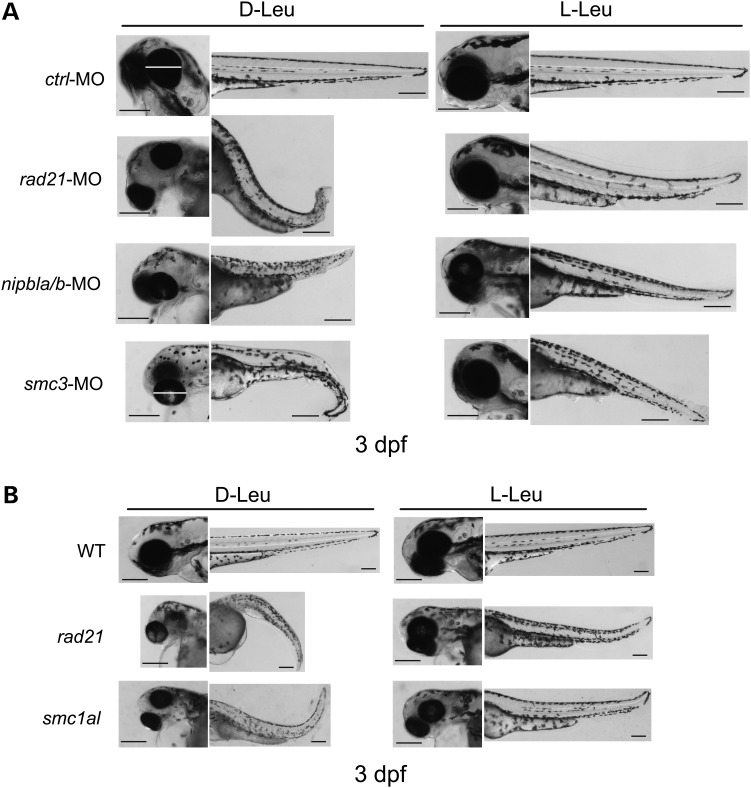
l-leucine supplement partially rescued the development of cohesin morphants and genetic mutant embryos. (**A**) Embryos were microinjected as in Figure 1, and the morphant embryos were supplemented with 3 mm l-leucine (l-Leu) or inactive d-leucine (d-Leu) plus 1 mm l-Glu following injection. The medium was replaced each day to maintain amino acid levels and avoid contamination. After 3 dpf, the embryos were photographed. The images show the positive effects of l-leucine on general development of embryos with *rad21*, *nipbla/b* and *smc3* depletion. While the image is representative, ∼240 embryos were observed per group. Scale bar = 200 µm. The number of severely defective and dead morphants were quantified (Supplementary Material, Fig. S4). (**B**) The *rad21* and *smc1al* transgenic mutant embryos were collected and supplemented with 10 mm l-leucine (l-Leu) or inactive d-leucine (d-Leu). After 3 dpf, the embryos were photographed. Scale bar = 200 µm. While the image is representative, ∼60 embryos were observed per group.

The cohesin morphants have multiple developmental defects such as small head and eyes, cardiac edema, shortened body length and curved tail (Figs. 1 and 2), similar to previous reports (13,14,16). The morphant phenotypes were persistent up to 5 dpf. Previous studies have used morpholinos to knockdown cohesin genes for 1–5 days (14,16). The *rad21* and *smc1al* transgenic mutant embryos exhibit phenotypes consistent with the cohesin morphant embryos at 1 to 5 dpf (Fig. 2 and data not shown).

**Figure 3.**
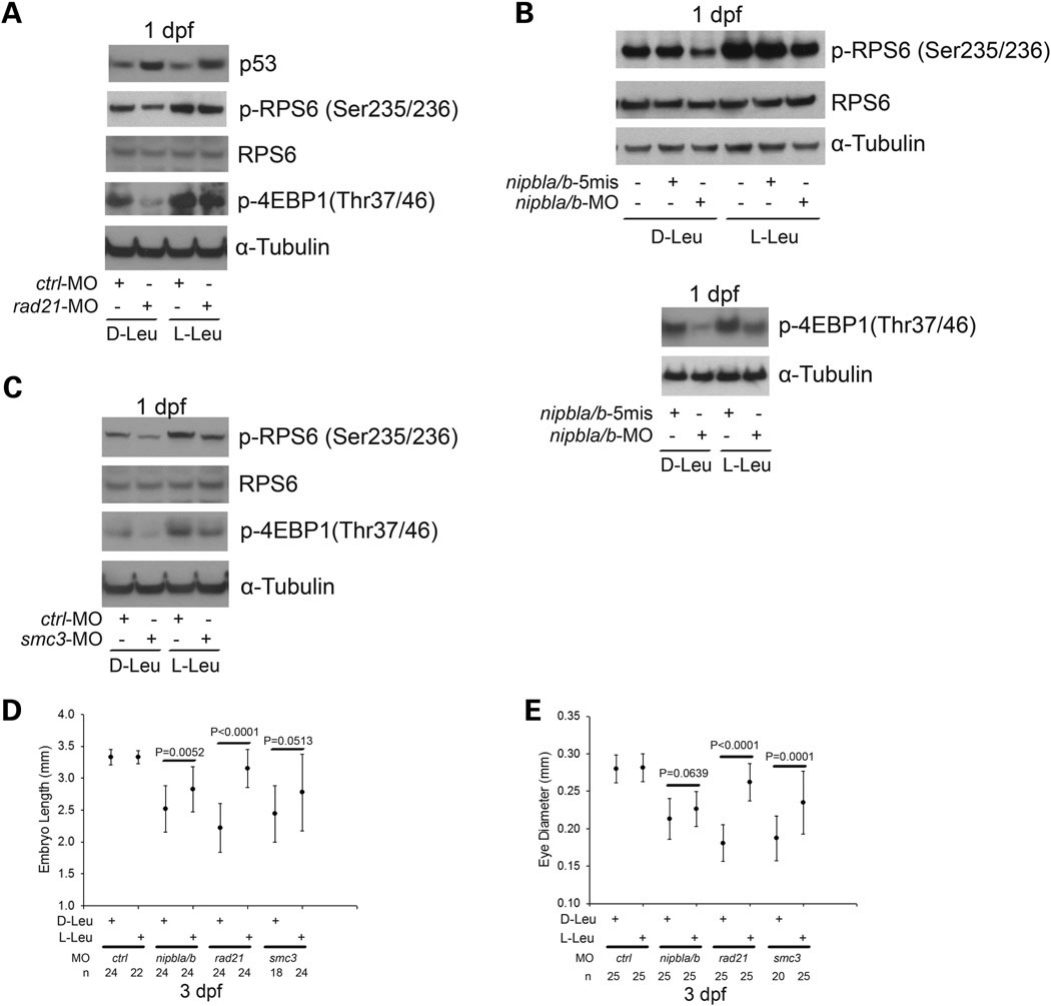
l-leucine treatment improved the molecular translational signature and partly rescued embryo length and eye size of cohesin morphants. (**A**–**C**) The morphant embryos were cultured as in Figure 2. After 1 dpf, we performed western blot analysis to observe phosphorylation of RPS6 and 4EBP1. Protein expression was quantified with ImageQuant TL software (Supplementary Material, Fig. S3). We drew a white line to show where the measurements were made for body length and eye size in Figures 1 and 2. (**D**) Morphant embryos were treated as in Figure 2. We measured embryo length for *rad21*, *nipbla/b* and *smc3* morphants with d-Leu or l-Leu treatment at 3 dpf. (**E**) Eye diameter of *rad21*, *nipbla/b* and *smc3* morphants with d-Leu or l-Leu treatment was measured. The number of embryos is indicated for each group.

To gain insight into the molecular signatures underlying the developmental defects in the cohesin morphants, we performed western blot analysis to examine p53 and RPS6. The p53 levels increased by ∼2-fold in the *rad21* and *smc3*-morphant embryos compared with *ctrl*-MO-injected embryos (Fig. 1B and Supplementary Material, Fig. S2). We have included a positive control for embryos exposed to ionizing radiation (10 Gy irradiation) and a negative control without any MO injection. The results showed that the p53 protein level was not upregulated in *nipbla/b* morphants compared with the uninjected embryos and *ctrl*-MO morphants, similar to p53 mRNA expression in a previous report (14). p27, also known as cyclin dependent kinase inhibitor 1b, is another protein that can slow the cell cycle. p27 was also elevated by ∼2-fold in the *rad21* and *smc3*-morphant embryos, which is suggestive of defective cell cycle progression. Western blotting results in all three morphants were consistent with translational impairment, demonstrated by reduced levels of the phosphorylated form of RPS6. Phosphorylation of RPS6, which is a downstream biomarker for the TOR pathway, promotes translation, cell growth and proliferation. Also, we observed that phosphorylation of 4EBP1 was reduced by more than half in *rad21*, *nipbla/b* and *smc3*-morphants compared with controls (Fig. 3A–C and Supplementary Material, Fig. S3A–C). 4EBP1 directly interacts with eIF4E, which is a limiting component of the multi-subunit complex that recruits 40S ribosomal subunits to the 5′ cap of mRNAs. When 4EBP1 is phosphorylated, the interaction with eIF4E is reduced, releasing eIF4E to initiate translation. The unphosphorylated form will repress translation initiation. Thus, the results suggested that CdLS morphant embryos have impaired translation.

As p53 was upregulated in *rad21* and *smc3* morphants, we tried to rescue the morphants with p53-MO. We selected a dosage (2 ng) that avoided toxicity. Depletion of p53 has been shown to rescue development in a Tcof mutant that had reduced ribosome function (24). However, we did not observe any substantial rescue of the *rad21, smc3* or *nipbla/b* morphant embryos with depletion of p53 (data not shown), similar to previous studies with the *esco2* morphants in which rescue was minimal (16,17).

### l-leucine treatment improves development and translation in cohesin-depleted embryos

l-leucine partially rescues molecular and developmental phenotypes of RBS zebrafish embryos by enhancing protein translation and cell growth through the mTOR pathway (17). We wondered whether l-Leu treatment could have similar benefits for the CdLS mutants and morphants. To explore the question, we cultured zebrafish embryos in 0.5XE2 media with l-Leu or inactive d-leucine (d-Leu) plus 1 mm of l-glutamine (l-Glu). l-glutamine promotes the transport of other amino acids (25). The cohesinopathy morphant embryos and the transgenic mutant embryos treated with l-Leu showed a remarkable improvement for several developmental deficiencies compared with those raised with d-Leu treatment (Fig. 2). We conducted morphological analysis at 3 dpf because distinct organs are easily observable and most cohesin morphants were still alive. l-leucine-treated embryos exhibited improved head and eye size, and more developed bodies with improved trunk length. We quantified the l-Leu effects on both severe developmental defects at 3 dpf and morphants' survival at 5 dpf (Supplementary Material, Fig. S4). The approximate number of embryos per group was 240. Severe malformation (representative examples shown in Figs 1A and 2) occurred in over 70% of untreated cohesin morphants but fell to 20–30% with l-Leu treatment. At 5 dpf, at least 60% of the untreated cohesin morphants died, compared with <40% with l-Leu. l-Leu treatment partially alleviates severe deformation and lethality in the cohesin morphant embryos.

We further quantified the effects of l-leucine on embryo length and eye diameter. We measured embryo body length from head to tail without taking body curvature into account. Cohesin morphant embryos showed poor growth as indicated by shortened embryo length (Fig. 3D). l-Leu supplement significantly improved *rad21*- and *nipbla/b*-MO embryo length (*rad21*, *P* < 0.0001; *nipbla/b*, *P* = 0.0052), while moderately promoting *smc3*-MO embryo length (*P* = 0.0513). Embryo eye diameter is severely compromised by cohesin knockdown. l-Leu treatment showed a range of rescue for eye size (Figs. 2A, 3E and 5A). The eye diameters of *rad21*- and *smc3*-morphants were significantly restored with l-Leu supplement (*rad21*, *P* < 0.0001; *smc3*, *P* = 0.0001), whereas *nipbla/b*-morphants were mildly improved (*P* = 0.0639).

**Figure 4.**
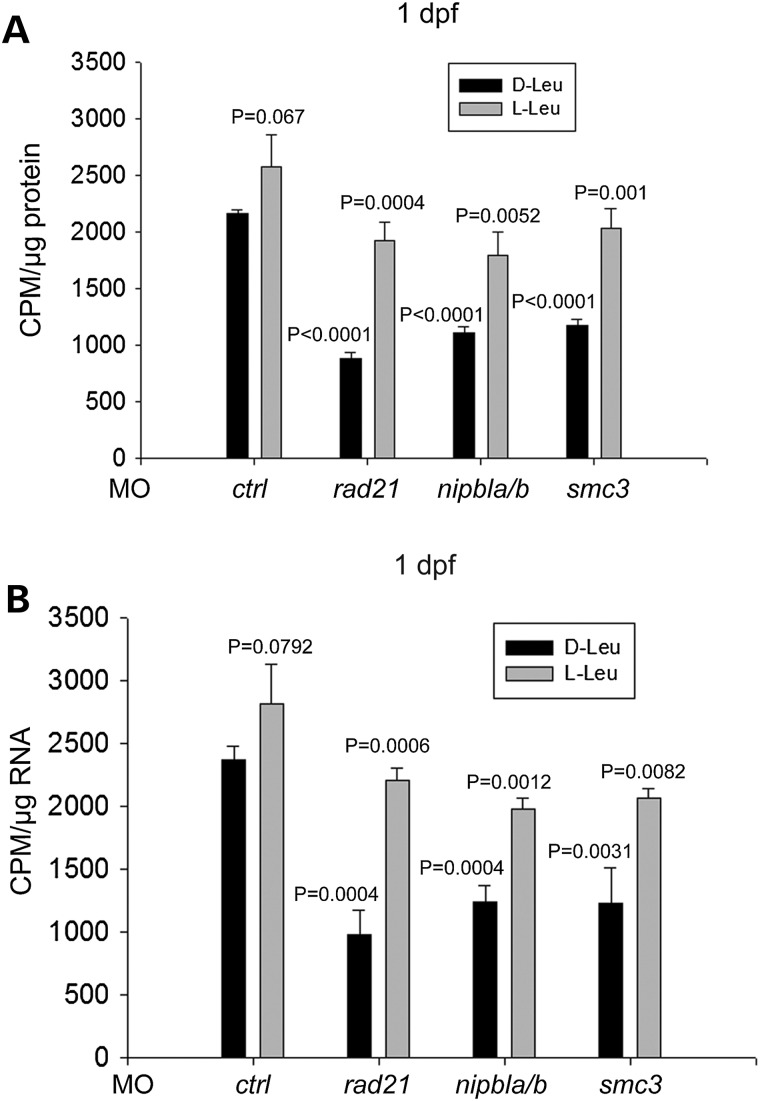
Supplementation of l-leucine partially boosted protein biosynthesis and rRNA production in cohesin-depleted embryos. (**A**) Morphant embryos were treated as in Figure 2. At 1 dpf, the embryos were collected to measure protein synthesis. ^35^S-Met labeling showed that l-Leu partly rescued protein synthesis in the cohesin morphant cells. Each bar represents the average ± SEM of ^35^S-methionine incorporation, as calculated for three independent samples. Incorporation was normalized to protein concentration. *P* = 0.067, *ctrl*-MO + d-Leu versus *ctrl*-MO + l-Leu; *P* < 0.0001, *rad21*-MO + d-Leu, *nipbla/b*-MO + d-Leu, *smc3*-MO + d-Leu versus *ctrl*-MO + d-Leu; *P* = 0.0004, *rad21*-MO + d-Leu versus *rad21*-MO + l-Leu; *P* = 0.0052, *nipbla/b*-MO + d-Leu versus *nipbla/b*-MO + l-Leu; *P* = 0.001, *smc3*-MO + d-Leu versus *smc3*-MO + l-Leu. (**B**) Morphant embryos were treated as in Figure 2. At 1 dpf, the embryos were collected to measure rRNA production. ^35^H-uridine incorporation showed that l-Leu partly improved rRNA biogenesis in the cohesin morphant cells. Each bar represents the average ± SEM of the ^35^H-uridine incorporation, as calculated for three independent samples. Incorporation was normalized to RNA concentration. *P* = 0.0792, *ctrl*-MO + d-Leu versus *ctrl*-MO + l-Leu; *P* = 0.0004, *rad21*-MO + d-Leu, *nipbla/b*-MO + d-Leu versus *ctrl*-MO + d-Leu; *P* = 0.0031, *smc3*-MO + d-Leu versus *ctrl*-MO + d-Leu; *P* = 0.0006, *rad21*-MO + d-Leu versus *rad21*-MO + l-Leu; *P* = 0.0012, *nipbla/b*-MO + d-Leu versus *nipbla/b*-MO + l-Leu; *P* = 0.0082, *smc3*-MO + d-Leu versus *smc3*-MO + l-Leu.

Immunoblotting analysis showed that l-Leu treatment boosted phosphorylation of RPS6 and 4EBP1 (Fig. 3A–C and Supplementary Material, Fig. S3). This pattern indicates that biomarkers of translation were partially rescued by l-Leu supplement in the cohesin morphants. However, p53 elevation persisted even with l-Leu treatment (Fig. 3A). As translation is tightly coupled with cell growth and proliferation, the results suggest that l-Leu supplement partly rescues the development defects of zebrafish CdLS embryos by strengthening translation. To directly test that protein translation is indeed affected by cohesin depletion, we performed ^35^S-methionine metabolic labeling to measure protein biosynthesis and ^3^H-uridine labeling to measure rRNA production in the cohesin morphants with and without l-Leu supplement at 1 dpf (Fig. 4). The cohesin morphant embryos display a reduction of global protein synthesis and rRNA biogenesis, which can be partly improved by l-Leu treatment. These results firmly establish that translation is defective in these embryos and can be improved with l-leucine.

**Figure 5.**
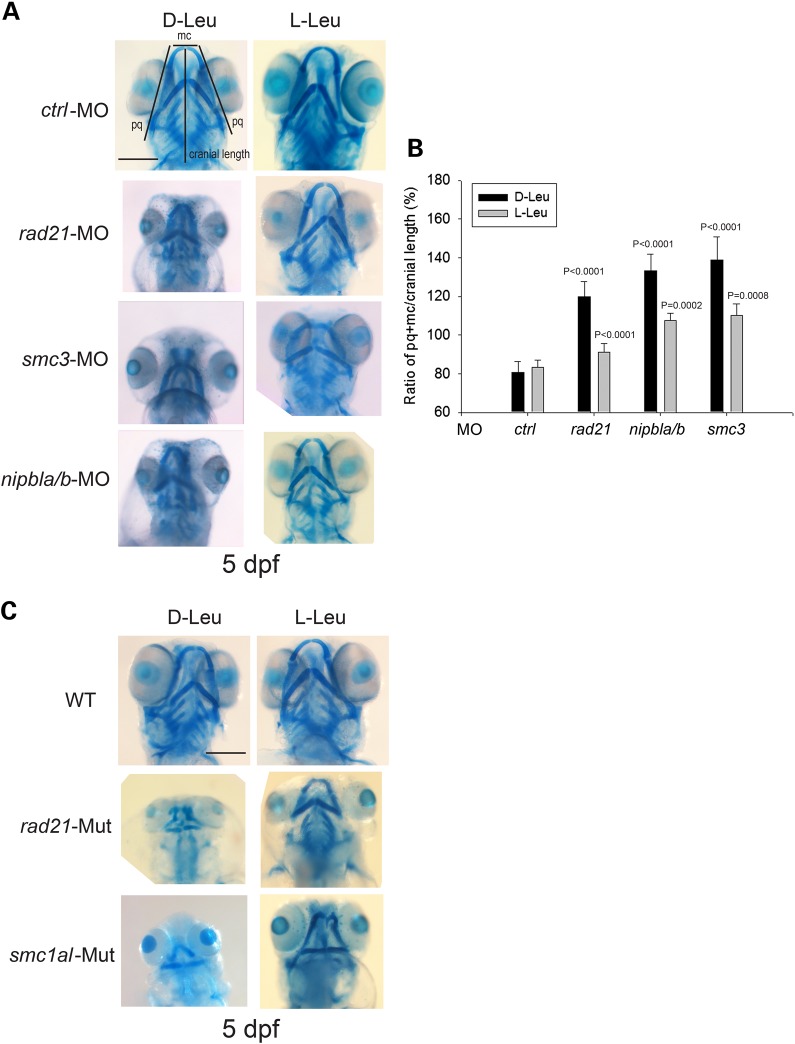
l-leucine treatment partially improved craniofacial cartilage development of cohesin morphants and transgenic mutant embryos. (**A**) Morphant embryos were cultured as in Figure 2. After 5 dpf, the embryos were stained with Alcian blue to detect cartilage development. Scale bar = 200 µm. While the image is representative, around 60 embryos were analyzed per group. (**B**) Cranial development was quantified for the data in (A) using the sum of the palatoquadrate (pq) cartilage and Meckel's cartilage (mc) divided by cranial length, as indicated with lines in (A). The measurement was done on eight embryos per group. *P* < 0.0001, *rad21*-MO + d-Leu, *nipbla/b*-MO + d-Leu, *smc3*-MO + d-Leu versus *ctrl*-MO + d-Leu; *P* < 0.0001, *rad21*-MO + d-Leu versus *rad21*-MO + l-Leu; *P* = 0.0002, *nipbla/b*-MO + d-Leu versus *nipbla/b*-MO + l-Leu; *P* = 0.0008, *smc3*-MO + d-Leu versus *smc3*-MO + l-Leu. Further quantification is shown in Supplementary Material, Figure S5. (**C**) The *rad21* and *smc1al* transgenic mutant embryos were cultured as in Figure 2. After 5 dpf, the embryos were stained with Alcian blue to detect cartilage development. Scale bar = 200 µm. While the image is representative, ∼40 embryos were analyzed per group.

CdLS is associated with craniofacial defects. Zebrafish models for CdLS have small head size and aberrant craniofacial morphology, suggesting some aspects of the human disease phenotype may be mimicked by the zebrafish model. We assessed craniofacial cartilage formation by staining with alcian blue at 5 dpf, a timepoint at which cartilage has been formed in the embryo. The *rad21*, *nipbla/b* and *smc3*-morphants as well as *rad21* and *smc1al* homozygous genetic mutant embryos displayed remarkably poor formation of craniofacial cartilage compared with control morphants and WT embryos. The morphants and mutants displayed partial rescue by l-Leu treatment (Fig. 5). The number of morphants with significantly improved cartilage formation was quantified (Supplementary Material, Fig. S5). The images in Figure 5A in the l-Leu column are representative of embryos classified as ‘improved.’ The rescue in the morphants was further quantified using the relative ratio of the sum of the palatoquadrate (pq) cartilage and Meckel's cartilage (mc) divided by cranial length, based on a method developed in a previous investigation (26). The rescue of the mutants was not further quantified because the cartilage formation with d-Leu was so poor that pq and mc could not be accurately measured. The images in Figure 5 also clearly show the increase in head size observed with l-leucine. Our results are consistent with a recent report in which defects in the P3K/Akt/mTOR pathway led to poor development of pharyngeal skeletal elements, which were partially rescued with l-leucine (27).

In contrast to the rescue of cartilage formation with l-leucine, the rescue of heart defects was very poor. Morphant embryos have been previously shown to have cardiac phenotypes including malformation (14,16) and a slow heartbeat (observation shown in supplement videos and quantification in Supplementary Material, Fig. S7). l-leucine provides only a mild rescue (10–20%) of the slow heartbeat at the gross level by normal brightfield stereoscope. Cardiac edema also persisted with l-Leu. Thus, while some aspects of development may be partially rescued by l-leucine, others are not. In the future, it will be interesting to further study how increasing translation with l-Leu affects specific cell types and development in the CdLS zebrafish model.

### l-leucine treatment improves cell proliferation in cohesin morphant embryos

**Figure 6.**
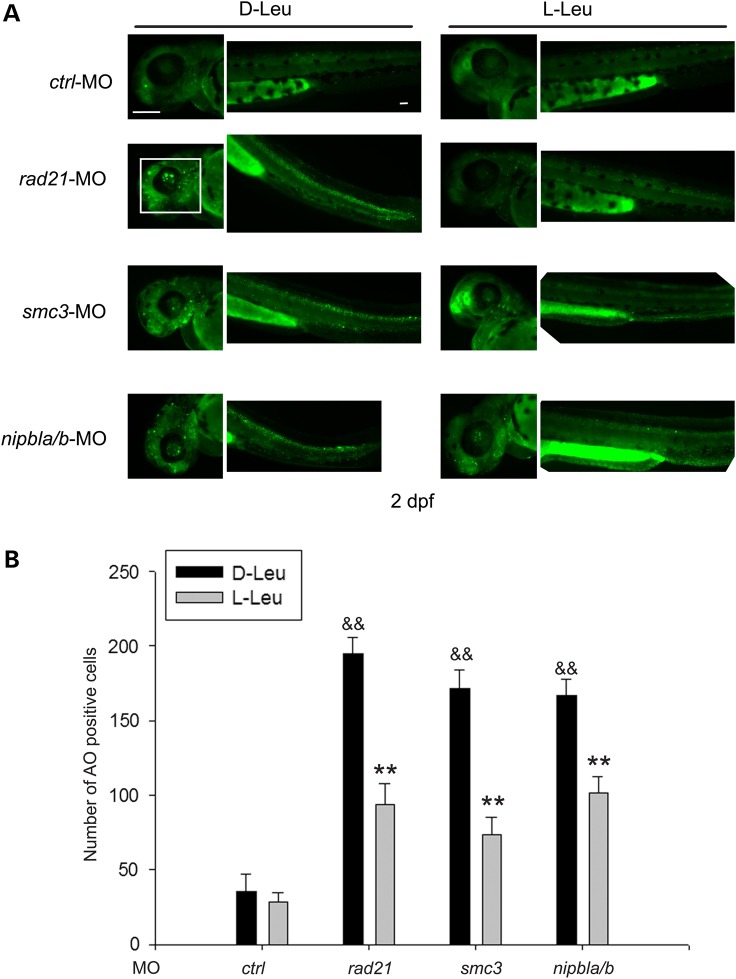
l-leucine partially prevents cell death in cohesin morphant embryos. (**A**) Morphant embryos were cultured as in Figure 2. After 2 dpf, the embryos were stained with acridine orange to detect dying cells. Scale bar = 200 µm. While the image is representative, ∼10 embryos were observed per group. (**B**). The number of AO-positive cells was quantified. Cell counts were done on images of a defined region of the head (white box) and tail of five embryos each. ^&&^*P* < 0.0001, *rad21*-MO, *nipbla/b*-MO or *smc3*-MO with d-Leu treatment versus *ctrl*-MO with d-Leu treatment; **P < 0.0001, *rad21*-MO with l-Leu treatment versus *rad21*-MO with d-Leu treatment, *nipbla/b*-MO with l-Leu treatment versus *nipbla/b*-MO with d-Leu treatment, *smc3*-MO with l-Leu treatment versus *smc3*-MO with d-Leu treatment.

Regulation of cell apoptosis is critical for embryonic development. The *esco2-*, *rad21-*, *nipbla/b-* and *smc3*-depleted embryos have all been reported to have a global increase in apoptotic cells by TUNEL assay and acute elevation of caspase3/7 activity (12,15–17,28). We performed acridine orange staining to look at apoptotic, damaged and necrotic cells in the morphant embryos. The zebrafish *rad21*, *nipbla/b* and *smc3* morphants all show at least a 4-fold increase in acridine orange–positive cells in the head and trunk. l-Leu treatment significantly reduced the number of acridine orange-positive cells in all three morphants (Fig. 6). l-leucine has been shown to boost translation activity, leading to a rise in cell growth and survival (29). Our results are consistent with the conclusion that some fraction of cell death is due to poor translation function in the cohesin morphants.

**Figure 7.**
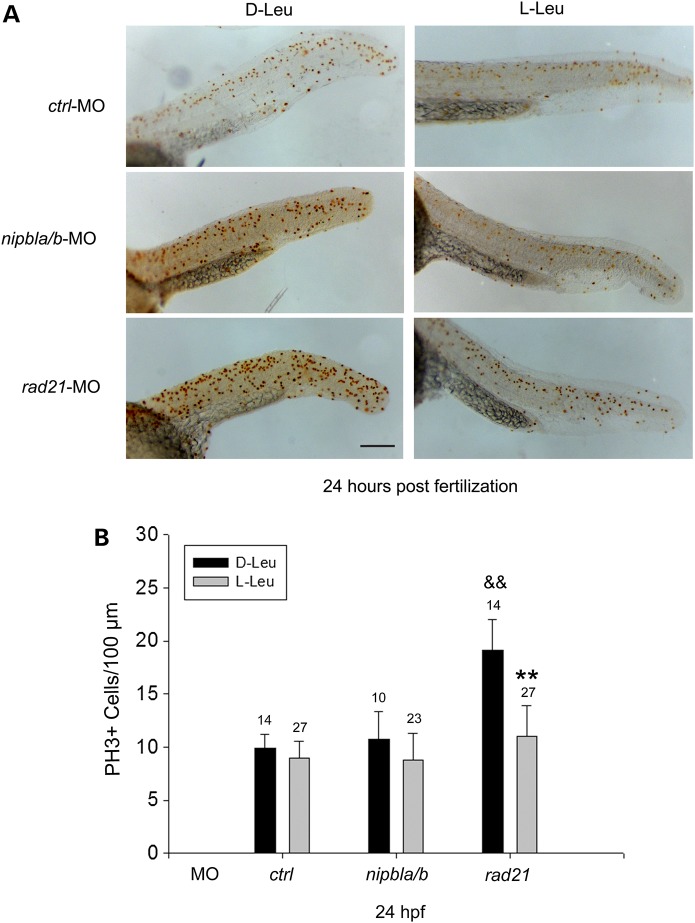
l-Leucine partially rescues high levels of pH3 in *rad21* morphant embryos. (**A**) Morphant embryos were cultured as in Figure 2. At 24 hpf, cells were immunostained with anti-pH3 antibody to detect mitotic cells. Scale bar = 200 µm. While the image is representative, 10–27 embryos were analyzed per group. (**B**) The number of cells in mitosis per µm^2^ was quantified for 10–27 embryos per group, with the embryo number indicated above the bar. ^&&^*P* < 0.0001, *rad21*-MO with d-Leu treatment versus *ctrl*-MO with d-Leu treatment; **P < 0.0001, *rad21*-MO with l-Leu treatment versus *rad21*-MO with d-Leu treatment.

Like cell death, cell division is tightly associated with protein biosynthesis. Thus, it is another vital factor that affects embryo development. The *rad21*-mutant embryos have an accumulation of G2/M cells compared with WT embryos (12). Mitotic delay was also observed in human RBS patient cells and zebrafish RBS morphants (16,17), which could potentially contribute to the slow growth phenotype of the embryos. Given the rescue effects of l-Leu on cohesin-depleted embryos, we wondered whether the mitotic delay could be overcome by l-Leu supplementation during embryo development. To answer this question, we immunostained cohesinopathy embryos with phospho-Histone H3 (pH3) antibody to identify G2/M cells in the presence of d-Leu or l-Leu, and the pH3-positive cells were quantified (Fig. 7). The *smc3*-morphants have not been reported to demonstrate elevation of pH3-positive cells, and therefore, we focused on the *rad21-* and *nipbla/b*-depleted embryos to measure the pH3-stained cells. Mitotic cells were elevated almost 2-fold (*P* < 0.0001) in *rad21*-morphant embryos and were significantly reduced by ∼50% (*P* < 0.0001) with l-Leu supplement. The *nipbla/b* morphants did not display an increase of pH3-positive cells. Collectively, this data indicate that l-Leu partially rescued mitotic delay in the *rad21*-depleted embryos. Interestingly, as p53 levels remain elevated with l-leucine treatment (Fig. 3), the rescue is independent of p53. mTOR signaling can control mitosis via the Chk1 kinase (30,31). This is one avenue by which l-leucine might rescue the mitotic delay, but other explanations are possible and the mechanism remains to be determined.

### The l-leucine metabolite alpha-ketoisocaproate (α-KIC) partially rescued development in *rad21*- and *nipbla/b*-MO embryos

l-leucine enhances both the TOR pathway and protein translation (32). We have previously tested whether other amino acids, such as l-glutamine alone, l-threonine, d-leucine and d-histidine, would rescue zebrafish RBS models and found limited rescue (17). High amounts of leucine supplementation can cause toxicity in zebrafish embryos. For instance, ∼10% of control embryos are dead at 5 dpf in l-leucine as compared with 5% in d-leucine (Supplementary Material, Fig. S4B). We wondered whether other related nutritional supplements could benefit cohesin-morphant embryos without the toxicity. Alpha-ketoisocaproate (α-KIC), a compound that can be converted to l-leucine via branched chain aminotransferase enzymes (33), also appears to activate TOR signaling and protein biosynthesis (21,22,34–37) and was therefore a good candidate.

**Figure 8.**
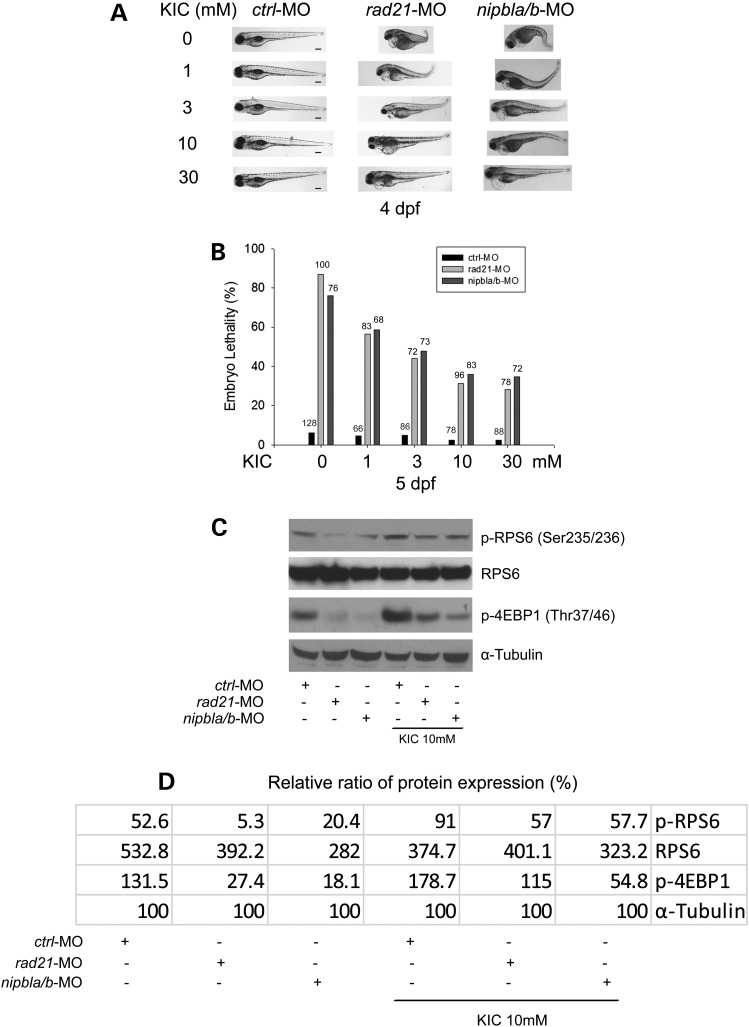
α-KIC partially rescues development, survival and the molecular signature of translational impairment in cohesin morphant embryos. (**A**) Embryos (1–2 cells) were microinjected with *ctrl*-MO or *rad21*-MO, *nipbla/b*-MO and immediately separated for α-KIC incubation in a concentration-dependent manner (0, 1, 3, 10 and 30 mm). The medium was replaced each day. At 4 dpf, these morphants were photographed to observe α-KIC effects. Scale bar = 200 µm. While the image is representative, ∼80 embryos were observed per group. Larger images are shown in Supplementary Material, Figure S6. (**B**). Embryo lethality was quantified at 5 dpf with α-KIC incubation. Total embryo numbers are indicated on each column. (**C**) Morphant embryos were incubated with or without 10 mm α-KIC. After 1 dpf, we performed immunoblotting analysis to examine RPS6 and 4EBP1 phosphorylation in *rad21-* and *nipbla/b*-depleted embryos with or without α-KIC treatment. (**D**) Protein expression from (C) was quantified with ImageQuant TL software.

**Figure 9.**
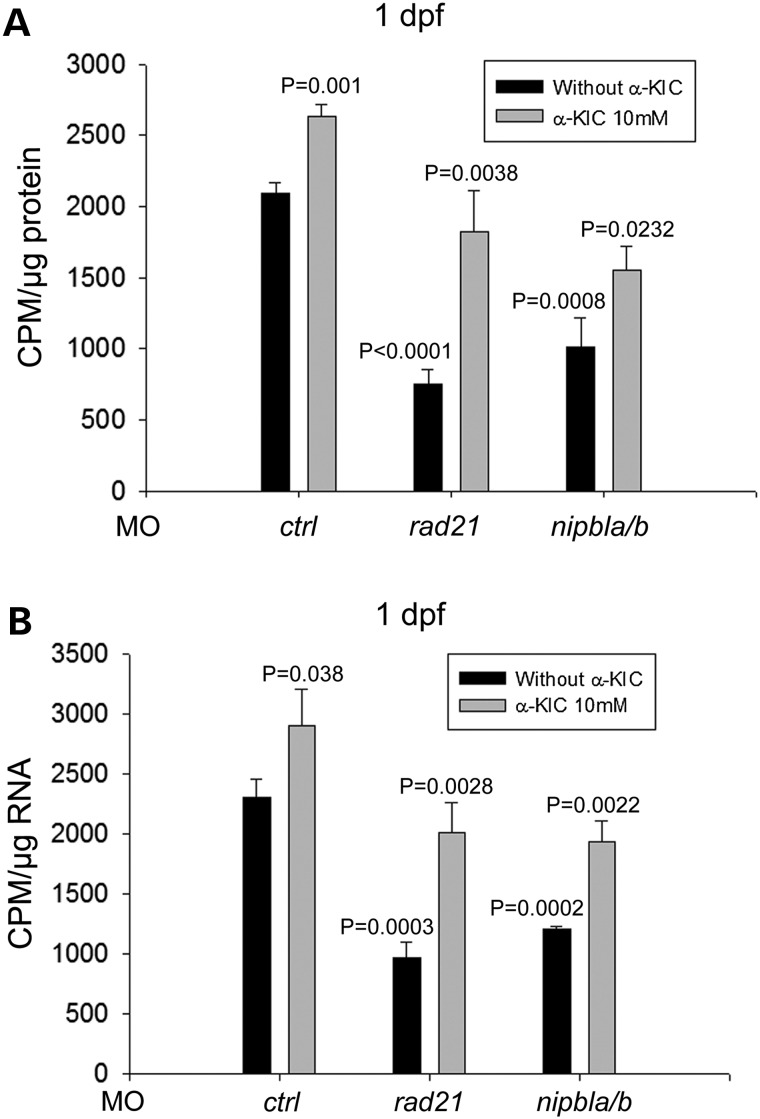
Supplementation with α-KIC partially boosted protein biosynthesis and rRNA production in the *rad21* and *nipbla/b* morphant cells. Morphant embryos were treated as in Figure 8C. (**A**) At 1 dpf, the embryos were collected to measure protein synthesis. ^35^S-Met incorporation showed that l-Leu partly rescued protein synthesis in the cohesin morphant cells. Data are presented as in Figure 4A. *P* = 0.001, *ctrl*-MO versus *ctrl*-MO + α-KIC; *P* < 0.0001, *rad21*-MO versus *ctrl*-MO; *P* = 0.0008, *nipbla/b*-MO versus *ctrl*-MO; *P* = 0.0038, *rad21*-MO versus *rad21*-MO + α-KIC; *P* = 0.0232, *nipbla/b*-MO versus *nipbla/b*-MO + α-KIC. (**B**) At 1 dpf, the embryos were collected to measure rRNA production. ^35^H-uridine incorporation showed that l-Leu partly improved rRNA biogenesis in the cohesin morphant cells. Data are presented as in Figure 4B. *P* = 0.038, *ctrl*-MO versus *ctrl*-MO + α-KIC; *P* = 0.0003, *rad21*-MO versus *ctrl*-MO; *P* = 0.0002, *nipbla/b*-MO versus *ctrl*-MO; *P* = 0.0028, *rad21*-MO versus *rad21*-MO + α-KIC; *P* = 0.0022, *nipbla/b*-MO versus *nipbla/b*-MO + α-KIC.

We examined the effect of α-KIC on development in the cohesinopathy embryos. The *rad21*- and *nipbla/b*-knockdown embryos were supplemented with α-KIC at 1 dpf in a concentration-dependent manner (0–30 mm) and replaced with 0.5× E2 media containing fresh α-KIC each day, up to 5 dpf. Imaging showed that α-KIC can partly rescue developmental defects in the cohesin morphants. However, α-KIC showed lower toxicity than l-leucine. The *rad21* and *nipbla/b* morphant embryos treated with α-KIC showed fewer development defects compared with those raised in embryo media without α-KIC (Fig. 8). Shortened body length and small head and eyes were less frequent in morphant embryos treated with α -KIC (Supplementary Material, Fig. S6). Furthermore, α-KIC promoted embryo survival in a dosage-dependent manner (Fig. 8B). In addition, western blotting showed that supplementing with α-KIC partially restored the phosphorylation of RPS6 and 4EBP1 in *rad21*- and *nipbla/b*-depleted embryos (Fig. 8C and D). Moreover, α-KIC also partially improved protein biosynthesis and rRNA production in *rad21* and *nipbla/b* morphant cells (Fig. 9). The data provide the first demonstration that translation can be boosted in an animal model with α-KIC.

**Figure 10.**
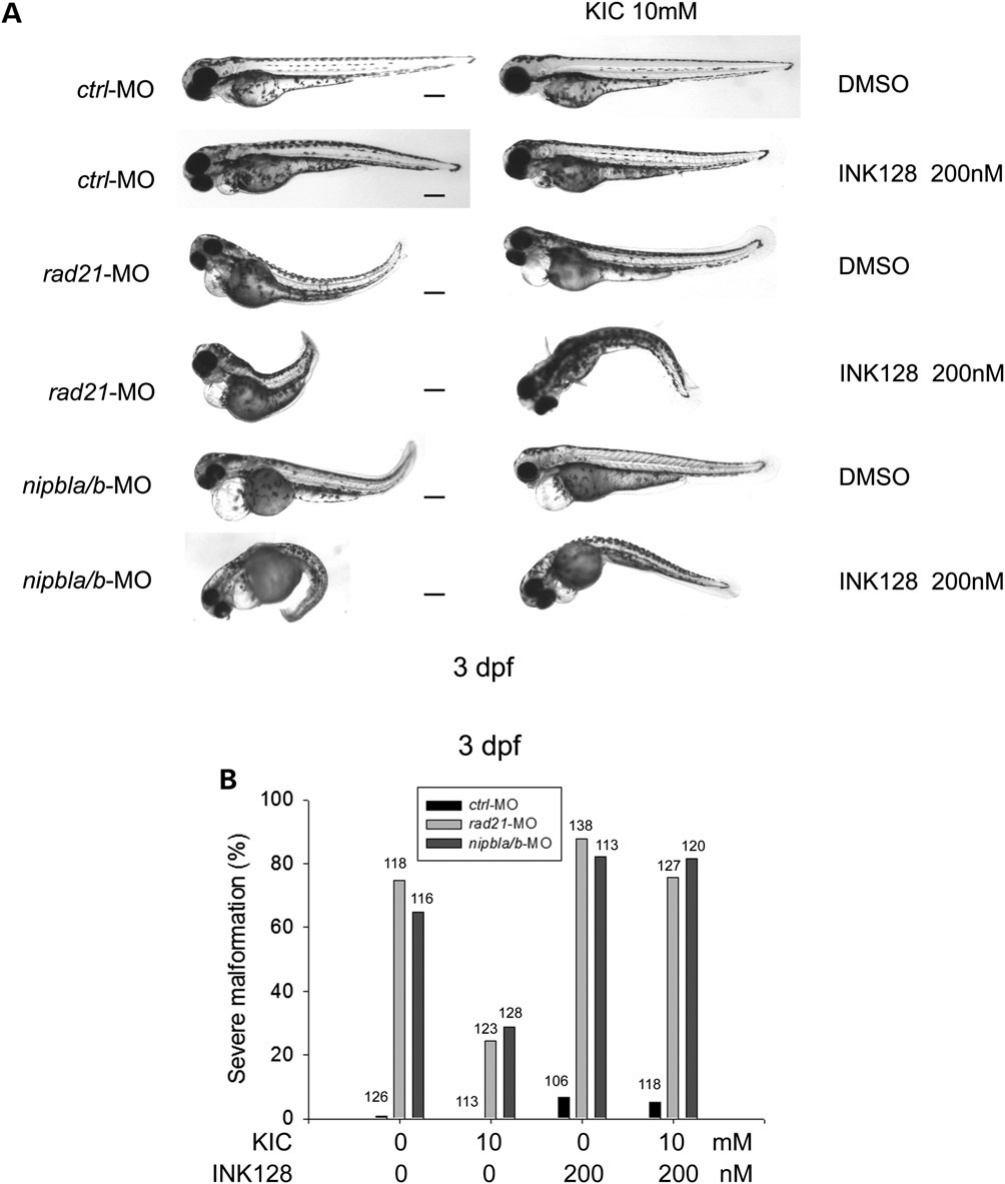
The mTOR inhibitor INK128 curtailed α-KIC rescue of *rad21* and *nipbla/b*-MO embryos. (**A**) Morphant embryos were generated as in Figure 8C and incubated in the presence or absence of INK128 (200 nm). The embryo medium was replaced each day. After 3 dpf, the morphant embryos were imaged. Scale bar = 200 µm. The inhibition of mTOR pathway with INK128 abolished rescue by α-KIC in *rad21* and *nipbla/b* morphants. (**B**) The percentage of severely defective morphants was quantified. The number of embryos in each group is indicated.

In order to establish that α-KIC is working via the mTOR pathway, we carried out the rescue experiment in the presence of an mTOR inhibitor INK128. We used a concentration of INK128 that generates slight defects in control embryos at 3 dpf (Fig. 10). INK128 curtailed the rescue by α-KIC in *rad21* and *nipbla/b* morphants (Fig. 10). The data indicate that α-KIC stimulation of the TOR pathway is able to partially improve development and survival in *rad21* and *nipbla/b* depleted embryos.

## Discussion

### Cohesin mutants share a defect in translation

Human cohesinopathies are caused by mutations in cohesin subunits, or regulators of the cohesin complex. In the zebrafish cohesinopathy models, we and others have observed that these different mutations share an overlapping spectrum of developmental defects including small size, curved trunks, poor craniofacial development, cardiac septal defects and disrupted blood circulation. The degree of these defects ranges from mild to severe. However, some molecular and cellular phenotypes are distinct. First, *esco2-*, *rad21-* and *smc3-*depleted embryos display p53 upregulation. In contrast, *nipbla/b* morphant embryos have a basal level of p53. The rescue effects of l-leucine appear to be independent of p53. Second, c-Myc mRNA expression is reduced in *rad21*, *nipbla/b* and *smc3* depleted embryos, in comparison with the upregulated expression observed in *esco2* mutants (13,14,16). Interestingly, c-Myc drives ribosome biogenesis and is a downstream target of mTOR (38), making it possible that one mechanism of rescue by l-leucine is through the increase in c-Myc function in *rad21-*, *nipbla/b-* and *smc3-*depleted embryos. Third, mitotic delay, which is detected with pH3 staining, occurs in *rad21* and *esco2* morphants, but not in *nipbla/b-* and *smc3*-depleted embryos. Mitotic delay appears to be rescued with l-leucine in the *rad21* and *esco2* morphants. Phenotypes that are shared by *rad21-*, *nipbla/b-*, *smc3-* and *esco2*-depleted embryos include elevated cell death and poor translation. Given the unifying feature of poor translation in cohesin morphant zebrafish embryos, it is not surprising that they all benefit to some degree from treatment with l-leucine to stimulate mTOR. *rad21* morphants showed the biggest defect in translation in this study and the most improvement with l-leucine or α-KIC treatment. Defective translation is also a shared feature of cohesin mutants in yeast and human cells. We speculate that an evolutionarily conserved function of the cohesin complex is to tie the chromosome structure to the translational output of cells. The mechanisms by which this is accomplished are likely related to the binding of cohesin at the rDNA and nucleolar function, but additional roles are possible and merit further investigation.

### Translation may be a productive therapeutic target for CdLS

Impaired translation and a positive response to l-leucine are shared features of zebrafish models of the cohesinopathies, including RBS and CdLS models. Impaired translation is also observed in another group of diseases known as the ribosomopathies, diseases attributed to defects in ribosome function. These diseases include Diamond-Blackfan anemia (DBA) (39) and 5q-syndrome (40), which both display hematological defects but also birth defects like craniofacial dysmorphology. The loss of ribosomal proteins (RP) in DBA and 5q-syndrome zebrafish models, while reducing protein translation, results in increased TOR pathway activity, as suggested by S6K and 4EBP1 phosphorylation (41). In zebrafish DBA models, l-leucine treatment further increased TOR pathway activity and partially rescued both development and anemia (41,42), independent of p53 (43). Two recent studies reported that different ribosomal subunit-deficient zebrafish embryos exhibited p53-independent defects, and treatment with the amino acid l-leucine resulted in a substantial rescue to the morphological defects and erythroid cells of these RP loss-of-function embryos (43,44). The results are reminiscent of our observation that l-leucine supplementation partially rescued morphological phenotypes of *nipbla/b* morphants, independent of p53 activity. Although the mechanism by which l-leucine stimulates the TOR pathway is under debate (45), oral l-leucine is currently being tested as a therapy in a phase I clinical trial in humans for DBA. Our results suggest that diseases with an underlying state of poor translation might in general be amenable to treatment with l-leucine.

Our study outlines some aspects of cohesinopathy zebrafish development that are partially rescued by l-leucine, suggesting they result from poor translation. These include embryo length, head and eye size and cartilage formation. However, not all developmental defects, including those in the heart, show rescue. A recent report showed that *nipblb* zebrafish morphants and CdLS patients fibroblasts displayed downregulation of the canonical Wnt pathway. Chemical activation of the pathway with lithium chloride (LiCl) partially rescued cell death and development in *nipblb*-morphant zebrafish embryos (28). LiCl has been shown to activate both Wnt and mTOR by an unidentified mechanism (46). Therefore, LiCl might rescue the morphant in part via its effect on translation. Now that reduced translation is established as a common feature of these animal models, it will be interesting to further explore how this defect contributes to development. l-leucine/α-KIC treatment will enable experiments that separate direct effects of cohesin on gene regulation from effects owing to reduced translation.

In conclusion, our study presents the first evidence in a non-yeast model that mutations in genes associated with CdLS affect translation. A previous study from our lab reported that mutations in Eco1, Smc1 and Scc2/Nipbl caused impaired translation in budding yeast (19). Our study implies that the relationship between cohesin function and translation has been conserved across several hundred million years of evolution. Our study further suggests that some of the developmental defects in the zebrafish models for CdLS are caused by translational defects, because treatment with l-leucine and α-KIC can partially rescue translation and development. Our work suggests that translation could be a productive therapeutic target in CdLS patients.

## Materials and Methods

### Ethics statement

All animals were handled in strict accordance with good animal practice as defined by the relevant national and/or local animal welfare bodies, and all animal work was approved by the Stowers Institute for Medical Research, Institutional Animal Care and Use Committee.

### Zebrafish maintenance and morphant embryo culture

Adult zebrafish were cared for by the Reptile & Aquatics Facility at the Stowers Institute for Medical Research. For microinjection, Stowers AB zebrafish were mated (1 male + 1 female per tank) to produce progeny. The embryos were washed once in embryo water and then kept in petri dishes until used for microinjection. Injected embryos were incubated in 3 and 10 mm d- or l-leucine and 1 mm Glutamine at 28.5°C directly after injection and followed up until 5 dpf. Every day, dead and live embryos were counted and live embryos were transferred to fresh l- or d-leucine media. By monitoring the amounts of live and dead embryos, statistics on survival were generated for each group and day.

### Morpholinos and microinjection

The *nipbl*, *smc3* and *rad21* morphants were generated by microinjection with antisense morpholino oligonucleotides (MO) obtained by GeneTools, LLC and compared with control-morpholino-injected embryos.

For *nipbl*, *rad21*, *smc3* and control (*ctrl*) morphants, 1 nl of morpholino solution diluted in Danieau's buffer was injected into the yolk of WT embryos at the 1- to 2-cell stage with a concentration of 0.5 ng/nl for *smc3*-MO, and 2 ng/nl for *nipbla/b*-MO, *rad21*-MO, *ctrl*-MO and *p53*-MO ensuring that the phenotype would not be lethal in the first few days but could be followed up until Day 5. To ensure proper injection, Phenol Red was added to the morpholino solution, which allowed visualization of the amount injected into the yolk.

Morpholino sequences obtained by GeneTools, LLC were:

*nipbla*-MO: 5′ ACGTGGACGCACAGGTTGCTCAGTG 3′
*nipblb*-MO: 5′ TGACGGCTGGGCACAGAAGTCTAAC 3′
*nipbla*-5mis: 5′ ACCTCGACGGACAGCTTCCTCAGTG 3′
*nipblb*-5mis: 5′ TGACGCCTCGGCAGAGAACTGTAAC 3′
*smc3*-MO: 5′ GTACATGGCGGTTTATGCACAAAAC 3′
*rad21*-MO: 5′ AGGACGAAGTGGGCGTAAAACATTG 3′
*p53*-MO: 5′ GCGCCATTGCTTTGCAAGAATTG 3′

As control, a random control Oligo-N MO (*ctrl*-MO) was used.

Embryos were followed up as described, and imaging was performed.

### Genotyping of *rad21* and *smc1al*-transgenic mutant embryos

For adult zebrafish, the Reptile & Aquatics Facility at the Stowers Institute for Medical Research performs fin clips and prepares DNA samples. For zebrafish embryos, genomic DNA was extracted, and genotyping was done by PCR analysis.

PCR primers are as follows:

*rad21*: hi2529
ctagcgtttggctaagcagctg
GCTCGCGCTGCTTACGTGCTG
Gccaaacctacaggtggggtc

The insert-bearing allele will give a 185-bp band, and the WT allele will give a 240-bp band.

*smc1al*: hi1113a
ctgcaggattactgcgtccg
ccatgctgaaccacttacttcaagg
gttccttgggagggtctcctc

The insert-bearing allele will give an 85-bp band, the WT allele will give a 265-bp band.

### Imaging

For length measurements as well as for the evaluation of the phenotype, imaging was performed using a Leica Stereoscope, a Leica camera and Leica application suite software (AxioVision). On 2–5 dpf, images were taken of each morphant and each incubation type (*nipbla/b*, *rad21*, *smc3*, *ctrl* in d- and l-leucine each) at a magnification of 24.7× and scaled to enable length measurements. For measuring lengths, the AxioVision software was used and curvature of the embryo was not taken into account. For each group and day, the lengths of all embryos were averaged.

For pH3 staining, the pictures were all scaled to the same size using the AxioVision software and positive (= mitotic) cells were counted manually. For each embryo, the area in which cells were counted was measured using the AxioVision software. A mean ratio of cells per 0.1 mm² was then calculated for each strain and incubation type (d- versus l-leucine).

Morphants co-injected with a p53 morpholino were used for imaging of the phenotype as described earlier.

### ^35^S-Met incorporation assay

Fifty embryos per group at 1 dpf were collected and washed in PBS. Embryos were dissociated by pipetting them up and down in 0.25% trypsin for 10–20 min at 28°C. The dissociated cells were washed in PBS and incubated in DME medium without methionine or cysteine (Sigma–Aldrich) at 28°C for 30 min. The medium was removed, and the cells were incubated in DME containing 20 µCi of ^35^S-labeled methionine and cysteine (PerkinElmer) at 28°C for 30 min. The cells were washed twice in PBS and lysed in RIPA buffer (50 mm Tris, pH 7.2; 150 mm NaCl; 1% sodium deoxycholate; 0.1% SDS; 1% Triton X-100; 10 mm NaF; 1 mm Na_3_VO_4_). Proteins were precipitated by the addition of hot 10% trichloroacetic acid. After centrifugation, the precipitate was washed twice in acetone. The precipitate was dissolved in 100 µl of 1% SDS and heated at 95°C for 10 min. The concentration of each total protein sample was measured by NanoDrop Spectrophotometer. An aliquot of the SDS extract was counted in Ecoscint for ^35^S radioactivity in a liquid scintillation spectrometer to determine the amount of ^35^S-methionine incorporated into proteins, and the amount per sample was normalized by 1 µg of protein.

### Ribosomal RNA assay

Fifty embryos per group at 1 dpf were collected and washed in PBS. Embryos were dissociated by pipetting them up and down in 0.25% trypsin for 10–20 min at 28**°**C. The dissociated cells were washed in PBS and incubated in DME medium (Sigma–Aldrich) at 28**°**C for 30 min. The medium was removed, and the cells were incubated in DME containing 20 μCi of ^3^H-labeled uridine at 28**°**C for 2 h. Total RNA was isolated with TriZol reagent (Invitrogen, USA), and the concentration of each RNA sample was measured by OD_260/280_. Each sample was counted in a Beckman LS 6500 multipurpose scintillation counter to determine the amount of ^3^H-uridine incorporated, and the amount per sample was normalized by 1 µg of total RNA. Three independent cultures were labeled to derive the standard deviation.

### pH3 staining

pH3 staining was performed according to ‘Analysis of the Cell Cycle in Zebrafish Embryos’ (47). Morphant and control embryos were dechorionated on Day 1 post-fertilization and fixed rotating in 4% paraformaldehyde at 4°C overnight. After permeabilizing them in −20°C acetone for 7 min, the embryos were washed in water, followed by two washes in PBST. Blocking for 30 min in 1% BSA/PBST was followed by incubation with the pH3 antibody [Cell Signaling Phospho Histone H3 (Ser10) (D2C8) XP^®^ Rabbit mAb #3377] at 4°C overnight (concentration 1 : 300). After four washings in PBST for 15 min each, the embryos were incubated with the secondary antibody (donkey-anti-rabbit labeled with HRP) in 1% BSA/PBST for 2 h. Another washing step (4 × 15 min in PBST) was followed by incubation in DAB solution + H_2_O_2_ for 10 min at room temperature in the dark. After a final wash (3 × 10 min in PBST), the embryos were kept in 50% glycerol/PBST at 4°C until imaging was performed. The number of pH3-positive cells was quantified on a consistent field (end of yolk extension to end of tail in age-matched embryos) per 100 µm^2^.

### SDS–PAGE and western blot analysis

Sodium dodecyl sulfate–polyacrylamide gel electrophoresis (SDS–PAGE) was performed using NuPAGE Novex 4–12% Bis-Tris precast gels (Invitrogen). Western blotting was performed according to standard protocol using a nitrocellulose (Whatman, Protran) or PVDF (Millipore, Immobilon-*P*) membrane. The following antibodies were used: phospho-RPS6 (Ser235/236), phospho-4EBP1 (Thr37/46) (Life Technologies, Grand Island, NY, USA), p53, p27 (AnaSpec, Inc.), S6 (Santa Cruz Biotechnology, USA) and α-tubulin (Sigma, MO, USA). Secondary antibodies were HRP linked, anti-rabbit IgG (from donkey) and anti-mouse IgG (from sheep) (GE Healthcare, NA934V, NA931V and NA935V, respectively).

### Alcian blue staining for cartilage

Embryos were collected, and most of the liquid was removed. 1 ml of 2% PFA/1× PBS pH 7.5 was added to the embryos followed by nutation for 1 h. Embryos were washed 1 × 10 min with 100 mm Tris pH 7.5/10 mm MgCl_2_, followed by addition of 1 ml of 0.04% alcian/10 mm MgCl_2_ stain pH 7.5 and overnight incubation with nutation. Embryos were washed with 80% EtOH/100 mm Tris pH 7.5/10 mm MgCl_2_ for 5 min, then 50% EtOH/100 mm Tris pH 7.5 for 5 min, then 25% EtOH/100 mm Tris pH 7.5 for 5 min. 1 ml 3% H_2_0_2_/0.5% KOH was added. Open tubes were incubated for 10 min, followed by 2 × 10 min with 1 ml 25% glycerol/0.1% KOH. Bleach was rinsed out, and 1 ml 50% glycerol/0.1% KOH was added. Embryos were nutated 10 min followed by a wash with fresh 50% glycerol/0.1% KOH.

### Cell death analysis of zebrafish embryos

The morphants were stained with acridine orange at 2 dpf to detect dying cells, which show green fluorescent granulated spots. The number of dying cells was quantified on a consistent field (end of head extension to end of yolk, and end of yolk extension to end of tail in age-matched embryos) for five embryos per group. To compare cell counts between the samples, a two-sided *t*-test was used.

### Statistical analysis

The results are reported as mean values ± standard error (mean ± SE). Statistical analysis was performed by Student's *t*-test with SigmaPlot-Systat Software (Sigmaplot Software, Inc.). An ANOVA two-way model was used to compare continuous variables. A *P* value of <0.05 was considered statistically significant.

## Supplementary Material

Supplementary Material is available at *HMG* online.

## Funding

This work was supported by the Stowers Institute for Medical Research, the Cornelia de Lange Syndrome Foundation, the March of Dimes Foundation (#6-FY14-434) and the NCI (CA154499). Funding to pay the Open Access publication charges for this article was provided by the Stowers Institute for Medical Research.

## Supplementary Material

Supplementary Data
