# Supplementary material for: l-leucine partially rescues translational and developmental defects associated with zebrafish models of Cornelia de Lange syndrome
Source: Hum Mol Genet. 2014 Nov 6;24(6):1540–55. doi: 10.1093/hmg/ddu565 (PMC4351377; doi:10.1093/hmg/ddu565)
Supplement: Supplementary Data [file supp_24_6_1540__index.html]

l-leucine partially rescues translational and developmental defects associated with zebrafish models of Cornelia de Lange syndrome — l-leucine partially rescues translational and developmental defects associated with zebrafish models of Cornelia de Lange syndrome — Supplementary Data 

# l-leucine partially rescues translational and developmental defects associated with zebrafish models of Cornelia de Lange syndrome

## Supplementary Data

Supplementary Data

**Files in this Data Supplement:**

- Supplementary Data - Pdf file
- Supplementary Video 1 - mp4 file
- Supplementary Video 2 - m4v file
- Supplementary Video 3 - mp4 file
- Supplementary Video 4 - m4v file
- Supplementary Video 5 - m4v file
- Supplementary Video 6 - m4v file
- Supplementary Video 7 - m4v file
- Supplementary Video 8 - m4v file
- Supplementary Video 9 - m4v file
