# Supplementary material for: l-leucine partially rescues translational and developmental defects associated with zebrafish models of Cornelia de Lange syndrome
Source: Hum Mol Genet. 2014 Nov 6;24(6):1540–55. doi: 10.1093/hmg/ddu565 (PMC4351377; doi:10.1093/hmg/ddu565)

Supporting information

Supplement figures

Figure S1. The cohesin gene morpholinos knockdown target gene expression. Embryos were microinjected by *ctrl*, *nipbla/b*, *rad21*, and *smc3*-MO. At 1 dpf, the morphant embryos were lysed and this lysate was used for Western blot analysis to measure protein expressionin (A, B, C). The expression of Nipbla/b, Rad21, and Smc3 was reduced by more than half in these morphants.

Figure S2. Quantification of protein levels in the cohesin depleted embryos in Figure 1B. Protein levels were quantified by ImageQuant TL software. p53 and its downstream transcriptional target p27 were upregulated by ~2 fold in the *rad21* and *smc3* morphants compared to uninjected embryos and *ctrl*-MO embryos. Phospho-RPS6 levels were decreased by over 50% in the cohesin depleted embryos.

Figure S3. Quantification of protein levels in the cohesin morphant embryos in Figures 3A,B,and C. L-Leu supplement did not change p53 upregulation in *rad21*-depleted morphants. The phosphorylation of RPS6 and 4EBP1 was reduced by over half in these cohesin morphant embryos, which was elevated ~2 fold by L-Leu treatment.

Figure S4. The number of severely defective and dead embryos is reduced with L-leucine supplement. Morphant embryos were generated as in Figure 2. (A) The number of severely defective morphants were counted at 3 dpf, and the percentage was calculated. An example of “severely defective” is shown in the D-leu images in Figure 2. Total embryo number at each group was *ctrl*-MO+D-Leu, n=243; *ctrl*-MO+L-Leu, n=243; *nipbla/b*-MO+D-Leu, n=266; *nipbla/b*-MO+L-Leu, n=252; *rad21*-MO+D-Leu, n=248; *rad21*-MO+L-Leu, n=236; *smc3*-MO+D-Leu,

n=273; *smc3*-MO+L-Leu, n=242. Each bar represents the average ± SEM of the severely defective morphants, as calculated for three independent samples. P<0.0001, *ctrl*-MO+D-Leu *vs nipbla/b*-MO+D-Leu, *rad21*-MO+D-Leu, *smc3*-MO+D-Leu; P=0.001, *nipbla/b*-MO+D-Leu *vs nipbla/b*-MO+L-Leu; P=0.0001, *rad21*-MO+D-Leu *vs rad21*-MO+L-Leu; P=0.0003, *smc3*-MO+D-Leu vs *smc3*-MO+L-Leu. (B) After 5 dpf, dead morphant embryos were counted, and the percentage was calculated. Dead embryos become black. Total embryo number at each group was *ctrl*-MO+D-Leu, n=276; *ctrl*-MO+L-Leu, n=283; *nipbla/b*-MO+D-Leu, n=303; *nipbla/b*-MO+L-Leu, n=316; *rad21*-MO+D-Leu, n=288; *rad21*-MO+L-Leu, n=318; *smc3*-MO+D-Leu, n=283; *smc3*-MO+L-Leu, n=296. Each bar represents the average ± SEM of the dead morphants, as calculated for three independent samples. P=0.0002, *ctrl*-MO+D-Leu *vs nipbla/b*-MO+D-Leu; P=0.0001, *ctrl*-MO+D-Leu *vs rad21*-MO+D-Leu, *smc3*-MO+D-Leu; P=0.0136, *nipbla/b*-MO+D-Leu *vs nipbla/b*-MO+L-Leu; P=0.0007, *rad21*-MO+D-Leu *vs rad21*-MO+L-Leu; P=0.0044, *smc3*-MO+D-Leu vs *smc3*-MO+L-Leu.

Figure S5. Morphant embryos had improved cartilage formation with L-Leu as evaluated by Alcian blue staining. A high percentage of cohesin morphant show poor cartilage formation. The percentage is signficiantly reduced with L-Leu supplementation. The total embryo number in each group is shown on each bar.

Figure S6. Large images of the head and tail in *rad21* and *nipbla/b*-depleted embryos show the rescue with α-KIC treatment. The scale bar = 200 µm.

Figure S7. The *rad21* and *nipbla/b* morphants displayed a slow heartbeat rhythm and cardiac edema. With L-Leu supplement, a small number of the morphants (10 to 20%) appear to be improved for the slow heartbeat defect.

Supplement videos of hearts in the *rad21* and *nipbla/b* morphant embryos with or without L-leucine supplement.

Supplement figure 1

A

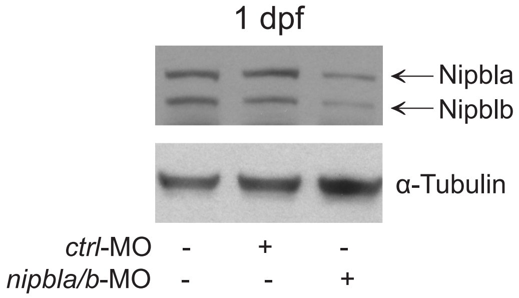

Relative ratio of protein expression (%)

22.8	23.1	8.2	Nipbla
18.6	16.1	4.1	Nipblb
100	100	100	α-Tubulin

MO - *ctrl* *nipbla/b*

B

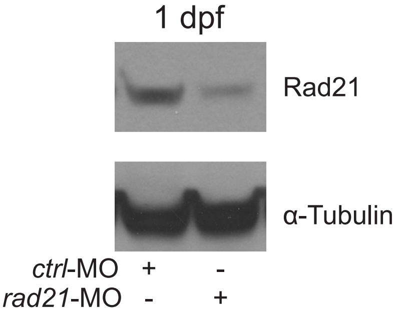

Relative ratio of protein expression (%)

26.7	9.2	Rad21
100	100	α-Tubulin

MO *ctrl* *rad21*

C

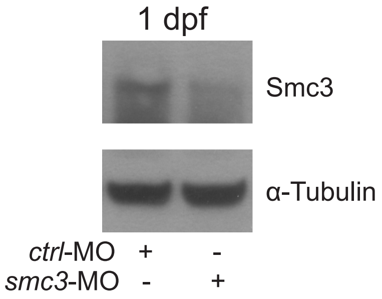

Relative ratio of protein expression (%)

31.7	15.7	Smc3
100	100	α-Tubulin

MO *ctrl* *smc3*

Supplement figure 2

Protein expression (%)

5.9	6	4.8	22.1	16.1	15.6	p53
55.6	53.2	55.1	97.8	98.6	108.4	p27
134.3	144.5	83.3	62.8	74		p-RPS6
58	44	57.1	59.1	55.6		RPS6
100	100	100	100	100	100	α-Tubulin
MO -	*ctrl*	*nipbla/b*	*rad21*	*smc3*	- Irradation	

Supplement figure 3

A

Protein expression (%)

28.5	58.1	30.9	59.5	p53
62.2	38.5	99.6	87.2	p-RPS6
18.8	18.1	18.5	19	RPS6
57.1	14.3	77.2	48.8	p-4EBP1
100	100	100	100	α-Tubulin
ctrl	*rad21*	*ctrl*	*rad21*	MO
D-Leu		L-Leu		

B

Protein expression (%)

274.4	237.6	126.8	306.8	324	277.7	p-RPS6
189.7	153.2	158.2	151.8	150.3	189.1	RPS6
100	100	100	100	100	100	α-Tubulin
uninjected	5mis	MO	uninjected	5mis	MO	*nipbla/b*
D-Leu			L-Leu			

Protein expression (%)

58.5	20.7	77.5	50.5	p-4EBP1
100	100	100	100	α-Tubulin
5mis	MO	5mis	MO	*nipbla/b*
D-Leu		L-Leu		

C

Protein expression (%)

23.8	11.8	49	34.9	p-RPS6
24.5	24.3	26	25.8	RPS6
41.4	20.8	87.7	68.3	p-4EBP1
100	100	100	100	α-Tubulin
ctrl	*smc3*	*ctrl*	*smc3*	MO
D-Leu		L-Leu		

Supplement figure 4

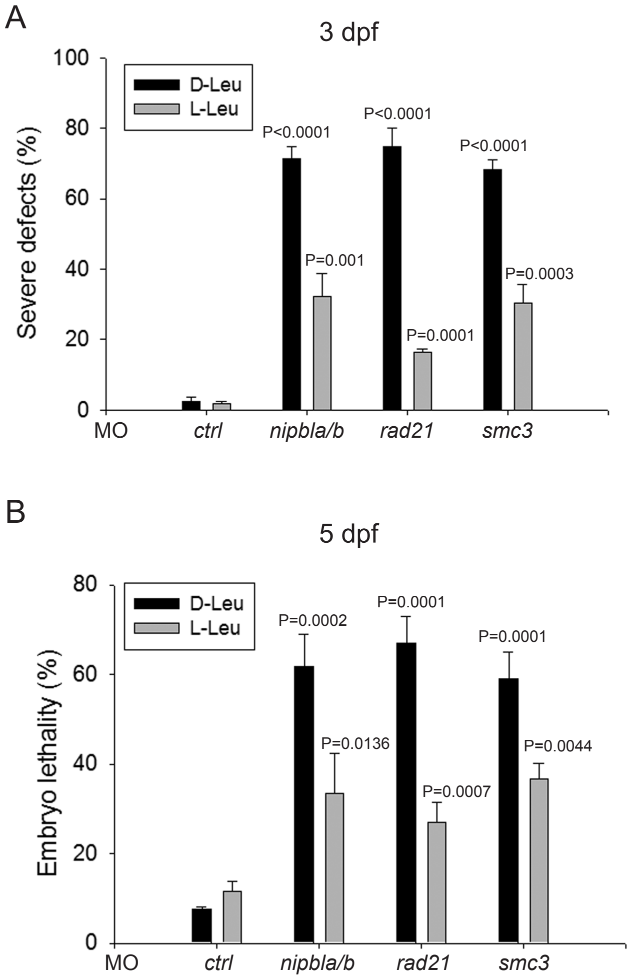

Supplement figure 5

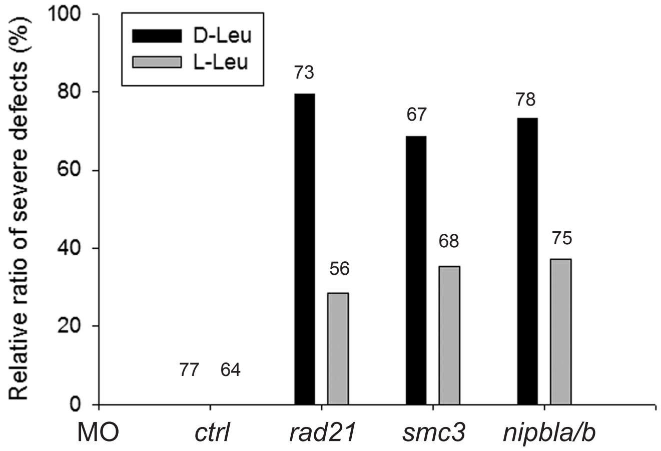

Supplement figure 6

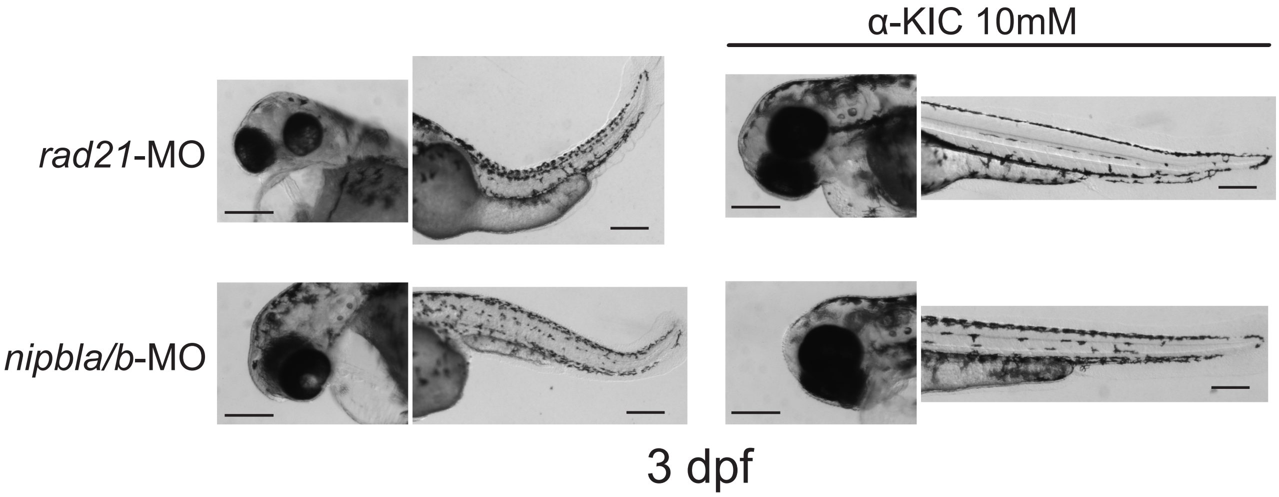

Supplement figure 7

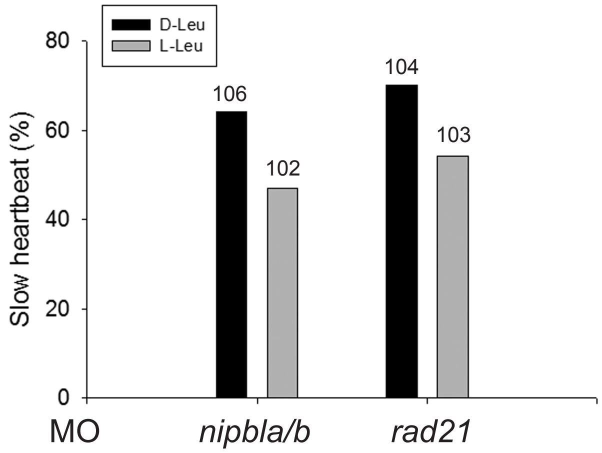

Supplement: Supplementary Data [file supp_ddu565_ddu565supp.pdf]
